# Infrared- and white-light retinal sensitivity in glaucomatous neuropathy

**DOI:** 10.1038/s41598-022-05718-6

**Published:** 2022-02-04

**Authors:** Grzegorz Łabuz, Asu Rayamajhi, Katarzyna Komar, Ramin Khoramnia, Gerd U. Auffarth

**Affiliations:** 1 The David J Apple Center for Vision Research, Department of Ophthalmology, University Hospital Heidelberg, Im Neuenheimer Feld 400, 69120 Heidelberg, Germany; 2 Institute of Physics, Faculty of Physics, Astronomy and Informatics, Nicolaus Copernicus University in Torun, Grudziądzka 5, 87-100 Toruń, Poland; 3 International Centre for Translational Eye Research, Skierniewicka 10A, 01-230 Warsaw, Poland; 4 Department of Physical Chemistry of Biological Systems, Institute of Physical Chemistry, Polish Academy of Sciences, Kasprzaka 44/52, 01-224 Warsaw, Poland

**Keywords:** Applied physics, Nonlinear optics, Translational research, Optic nerve diseases, Retinal diseases, Vision disorders

## Abstract

Glaucoma causes irreversible neuropathy, which, untreated, may lead to blindness. In this case–control study, we measured two-photon infrared (IR) light sensitivity in glaucomatous eyes to propose a new method to quantify the visual loss. In total, 64 patients were recruited with an equal distribution between glaucoma and control groups. Retinal sensitivity to IR light was assessed using a two-photon excitation device. A fundus-driven microperimeter was used to measure retinal sensitivity to visible light. The retinal nerve fiber layer (RNFL) thickness was quantified automatically with optical coherence tomography. The IR sensitivity of glaucoma and control eyes differed significantly (*P* = .003): 9.8 (6.5 to 13.1) dB vs. 10.9 (8.2 to 13.0) dB. Although in the visible-light microperimetry, retinal sensitivity was decreased in glaucoma (17.0, range: 6.9 to 20.0 dB) compared to the controls (17.7, range: 11.6 to 20.0 dB), this difference did not reach the significance level. A significant thinning of the RNFL in the glaucoma group was observed (*P* < .001). IR sensitivity significantly correlated with the RNFL in three of the four assessed quadrants instead of only one in visible-light microperimetry. Although further research is needed, this proof-of-concept study suggests that IR-light sensitivity can be used to support the detection of glaucomatous neuropathy.

## Introduction

Glaucoma is a multifactorial and progressive neurodegenerative disease and the third largest cause of irreversible visual loss^1,2^. An estimated 76 million glaucoma patients worldwide is projected to approach 112 million by 2040^1^. The onset of the disease can be imperceptible to the patient and challenging for the clinician to identify^1–3^.

Therefore, early detection of glaucoma is vital to reduce vision loss and to enable timely diagnosis. However, detection depends on screening methods—tonometry flags patients based on an elevated intraocular pressure (IOP), but its sensitivity may be limited^4^. Visual-field testing identifies areas of decreased visual function, and optic-nerve imaging techniques, such as optical coherence tomography (OCT) or confocal scanning laser ophthalmoscopy (SLO), provide an additional morphological aspect to patients' screening^1–3,5–11^. The structural changes of the optic nerve head and thinning of the retinal nerve fiber layer (RNFL) may be observed using imaging tools before the visual-field defects occur, i.e., pre-perimetric glaucoma^12^. However, the utility of this approach may be hampered by the variability of morphological parameters in the population. For instance, high-myopic eyes may have thinner retinal layers compared to those of emmetropic healthy eyes, which may be erroneously characterized as glaucomatous^13^. Also, the variability in healthy eyes' optic disc appearance poses challenges to diagnosis based (solely) on morphological assessment^14^. Hence, it is essential to combine functional and imaging approaches and evaluate their correlation in the detection of glaucomatous neuropathy^6^.

Fundus-driven perimetry (microperimetry) applies this principle and combines the assessment of retinal sensitivity, considering the patient's fixation behavior (real-time fundus-tracking) during the examination, and obtaining simultaneously retinal images^5–7,9,10^. This approach can quantify how structural changes at the retina translate into a functional effect^6^. A recently introduced two-photon excitation technique provides a new parameter of retinal function^15–19^. In contrast to standard (single-photon) vision^20^, the two-photon absorption by photoreceptor cells is a non-linear process that yields color perception while exposing the eye to infrared (IR) light^15–18^. For instance, a 1045-nm beam of light can be seen as green with the wavelength corresponding approximately to half of the stimulation beam (i.e., 522.5 nm)^15,18^. In laboratory studies, the two-photon excitation approach demonstrated a higher precision than visible-light technology in testing retinal function and resistance to lenticular opacification^16,21^. This novel approach has recently been applied in the studies of IR vision in the healthy population and patients with diabetic retinopathy^17,18^. However, the impact of glaucoma on IR-light sensitivity has not yet been evaluated.

Our study aimed to assess retinal sensitivity using a standard visible-light microperimetry and a novel two-photon excitation device in glaucoma patients and healthy volunteers and correlate functional results with OCT-derived morphological parameters.

## Methods

### Study population

**Figure 1:**
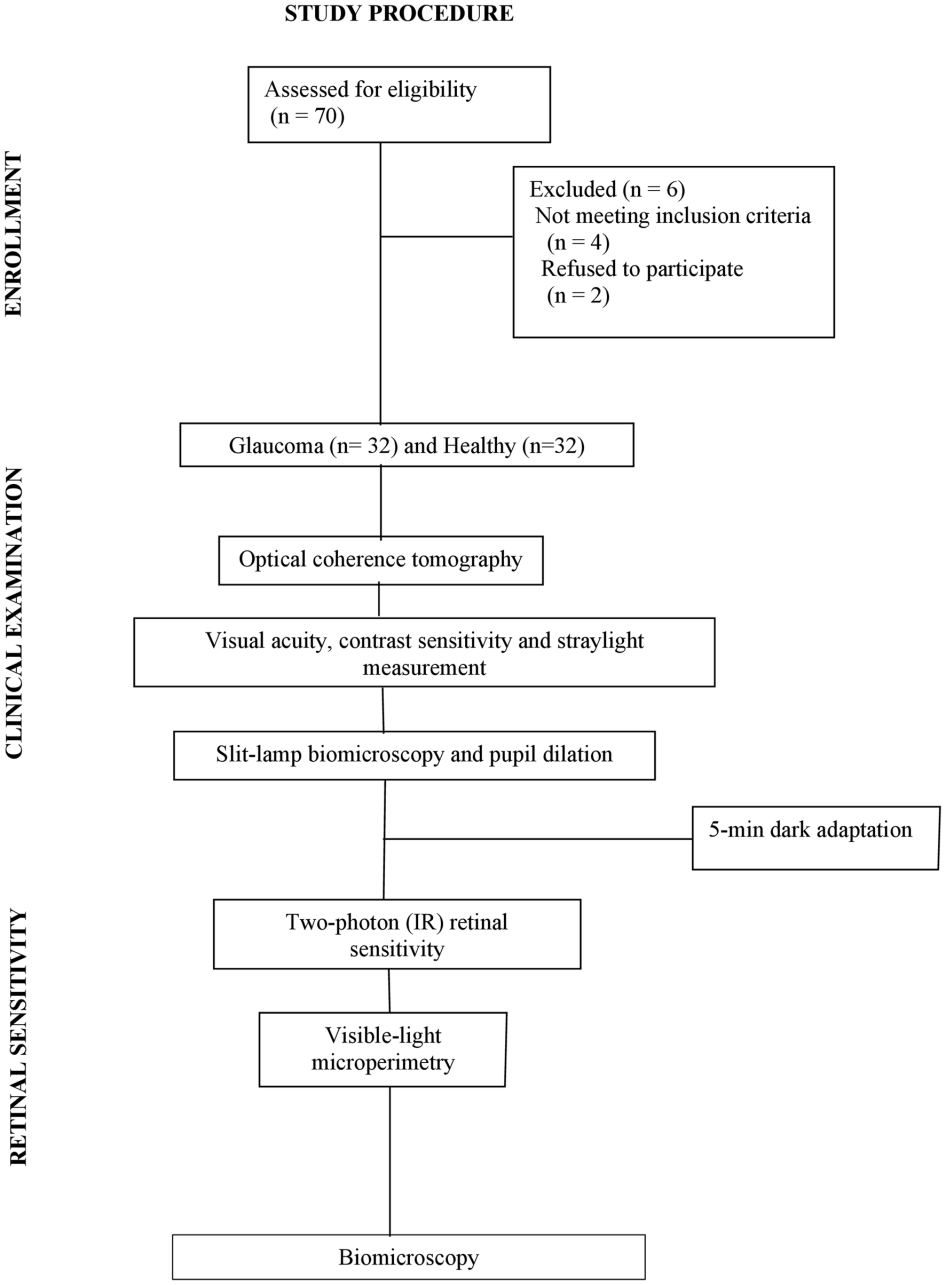
Flow chart of the study procedures.

This case–control study, which adhered to the tenets of the Declaration of Helsinki, was approved by The Ethics Committee of the Medical Faculty of Heidelberg University. Participants were recruited at the outpatient department of Heidelberg Eye Clinic and divided into two groups. The study group consisted of subjects diagnosed and treated for primary open-angle glaucoma, and a control group encompassed age-matched healthy volunteers. Glaucoma was confirmed by an experienced glaucoma specialist through a series of examinations based on optic-neuropathy characteristics, quantification of the RNFL, and visual-field defects. Glaucoma patients were recruited at their follow-up visits. All had a history of elevated IOP; thus, all were on intraocular-pressure-reducing medication at the time of their participation in the study. Candidates with any systemic or eye disease (other than glaucoma in the study group) or surgical antecedent (except cataract surgery) were deemed ineligible. Only eyes with uncompromised visual acuity (VA) equal or better than 0.10 logMAR were included in the control population. Each group's participant had to have the refraction's spherical component within ± 4.00 D and astigmatism lower than 1.50 D due to the limited refractive-error correction capabilities of the IR-sensitivity device. All recruits signed written informed consent after receiving a thorough explanation of the study. Figure 1 lists the procedures performed.

### Clinical examination

Prior to joining the study, participants underwent comprehensive ocular examination. Following the refractive-error correction using a trial frame, best-corrected VA was determined with Early Treatment Diabetic Retinopathy Study charts. Light scattering (straylight) was assessed using a C-Quant device (Oculus GmbH, Germany) which gives an objective measure of ocular turbidity. Population studies have shown that a normal young eye has, on average, 0.86 log(s). However, this parameter has shown a strong age dependence and a sharp increase over the age of 50 years with a threefold elevation at 77 years (1.31 log[s])^22^. In the pseudophakic eye, only a minimal, albeit significant, increase of straylight with age was observed with the mean value of 1.21 log(s) reported in the population^23^. After a complete straylight assessment, 5 mg/mL tropicamide (Mydriaticum Stulln; Pharma Stulln GmbH, Germany) was instilled for a subsequent retinal-sensitivity and slit-lamp evaluation. The Spectralis OCT (Heidelberg Engineering, Germany) was used for macular screening (30° scan angle) and to objectively measure the RNFL using a built-in layer-segmentation tool of the device.

### Retinal sensitivity assessment

Following the confirmation of patients' eligibility, a study eye was selected through randomization. The visibility threshold was measured (1) in visible light using an MP-1 microperimeter (Nidek Technologies Srl, Italy) and (2) in IR light using the two-photon excitation device. The choice of which test (visible or IR) to perform first was randomized.

The MP1 microperimeter is a commercial device that can assess the central visual field up to 22.5° in the visible range. Retinal sensitivity is measured through a built-in liquid crystal display projecting white-light stimuli. The dynamic range of sensitivity measurements spans from 0 dB (400 abs) to 20 dB (4 abs). The device's eye-tracker compensates for eye movements during testing. After each examination, a color fundus image (two-dimensional) is recorded using a non-mydriatic fundus camera with a 45° field of view. Sensitivity results are then overlaid on the fundus image, which enables a direct correlation between morphological changes at the retina and their functional effects.

**Figure 2:**
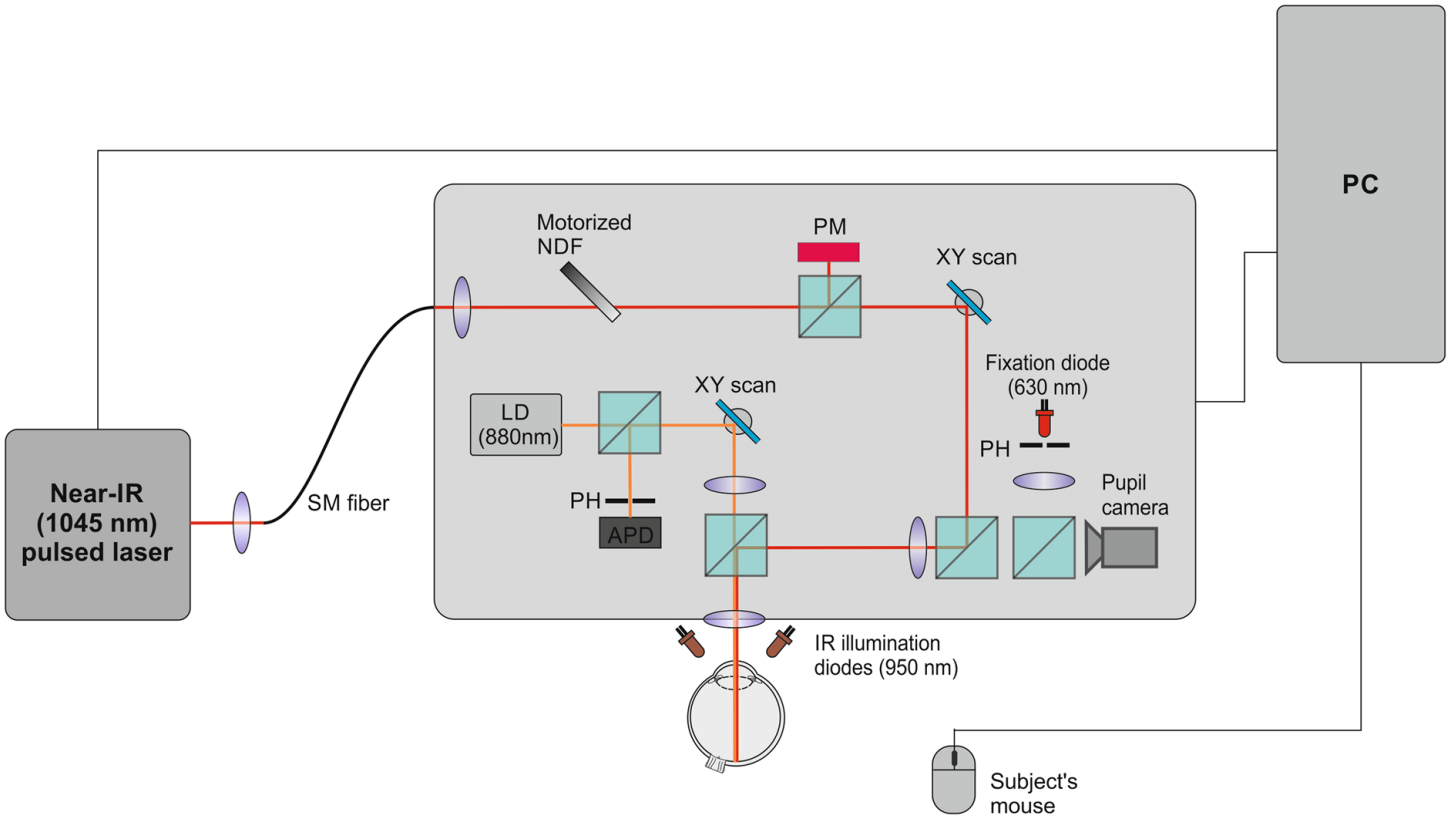
Block diagram of the the optical setup for infrared-light retinal-sensitivity measurements. λ = wavelenght; F = repetition frequency; $\tau$ = pulse width; SM = single mode; NDF = neutral density filter; PM = power meter; PH = pinhole; LD = laser diode; APD = avalanche photodiode; SLO = scanning laser ophthalmoscopy, PC = personal computer^18^.

The two-photon device we used is not yet commercially available. Figure 2 contains a schematic drawing of the setup. It employs a femtosecond laser, the HighQ-2 laser (Spectra-Physics, CA, USA), to project IR-light stimuli through ultrashort pulses with 63 MHz of the pulse-repetition frequency and 250-fs pulse width^17,18^. The monochromatic IR light has a wavelength of 1045 nm and a full width at half maximum of 8 nm. An array of neutral-density filters attenuates the laser power to meet the safety requirements of ANSI Z136.1-2014. The calculation of the maximum permissible exposure can be found in our earlier publication^17^. The minimum and maximum power was 1 µW (26 dB) and 400 µW (0 dB), respectively, which also defined the device's dynamic range.

Two-dimensional fundus visualization is enabled through integrated SLO. An IR camera for pupil preview is used for head position based on Purkinje images. Both the SLO and pupil-preview systems were used to monitor patients' compliance during the examination. The testing procedure resembles standard perimetry; thus, it is characterized by a comparable level of difficulty. First, a patient was seated with the head placed on an adjustable chinrest. A red-point target was projected on which the patient fixated during testing. After receiving a complete introduction to the measurement procedure, a pre-test was performed for the patient's training and to ensure that all instructions were clear and understood. A standard staircase procedure was used for the IR-stimuli projection with gradually increasing intensity until the visibility threshold was reached. Two measurements per retinal locus were performed in two measurement cycles with the randomized order of stimuli location. The examination time varied between 10 and 15 min depending on the patient’s ability and retinal sensitivity (i.e., it was extended in patients with large areas of decreased sensitivity).

Table 1 details the measurement conditions for IR- and visible-light testing. Those parameters were kept similar to enable a direct comparison between the two instruments.

**Table 1:** Comparison of the measurement conditions with the two devices.

	Two-photon (IR) device	Visible-light microperimeter
Fixation point	Red	
Stimulus size	Goldmann III	
Stimulus duration	200 ms	
Staircase strategy	Monotonous increase	4–2–1
Grid	12° (44 points)	
Dark adaptation	Yes (5 min)	
Background luminance	1.27 cd/m^2^	
Light spectrum	Monochromatic (IR)	Polychromatic (visible range)

### Statistical analysis

The sample size was estimated using G*Power software (Düsseldorf University)^24^ based on the recently published data on IR-light sensitivity, which showed the healthy patients' average of 15.5 ± 1.3 dB^17^. A type I error of 0.05 was used, and the statistical power was set at 0.80 in a two independent-sample t-test. In that case, 56 subjects would be needed to test the null hypothesis that IR-light sensitivity differs significantly between the control and glaucoma groups with a 1-dB threshold difference. The sample size was increased by 15%, giving a total of 64 subjects (32 per group) to account for the possible exclusion of cases.

MATLAB (Mathworks, Inc., NY, USA) and R (R Foundation for Statistical Computing, Austria) were used for data analysis and visualization. Retinal-sensitivity grids of the left eye were flipped along the vertical meridian to be analyzed and displayed as of the right eye. Due to the skewness of the visibility thresholds' distribution (particularly in the visible range) inferred from a Q-Q plot analysis, we applied non-parametric statistical methods. Still, the shape of each group's distribution was comparable. Mann–Whitney U-test was used for the comparison between two groups' mean values. Spearman's rank correlation coefficient (ρ) was calculated to evaluate the relationship between measure outcomes. Quantile regression was used in the comparison of the visible- and IR-light sensitivity. A 5% significance level was set. The median and the range summarized numerical data.

## Results

In total, 64 patients were enrolled in the study with an equal distribution between the study groups. The median (range) age of the healthy population was 65.4 (48.7 to 79.9) years, and the glaucoma patients were 65.3 (46.2 to 81.0) years of age. The difference did not reach a significance level (*P* = 0.82). The controls had significantly (*P* = 0.001) higher VA (− 0.05, − 0.18 to 0.24 logMAR) than the diseases patients (0.02, − 0.16 to 0.46 logMAR). No difference was noted in the subjects' spherical equivalent (control eyes: 0, − 3.75 to 2 D vs. glaucomatous eyes: 0, − 4.75 to 2.75 D, *P* = 0.93). Ocular turbidity level was also comparable (*P* = 0.0.19), as in the healthy population, we found 1.10 (0.90 to 1.48) logs and 1.16 (0.60 to 1.95) logs in the glaucoma patients. Table 2 shows the structural damage developed in the disease course resulted in a significant RNFL thinning observed in all quadrants.

**Table 2:** The retinal nerve fiber layer thickness measured in the control and glaucoma groups.

	Control			Glaucoma			*P*-value*
	Median [µm]	Min [µm]	Max [µm]	Median [µm]	Min [µm]	Max [µm]	
Inferior	122	58	146	83	45	141	< 0.001
Superior	113.5	62	159	73	27	134	< 0.001
Nasal	73	36	108	53	22	92	< 0.001
Temporal	74	54	131	53.5	35	85	< 0.001
Global	95	67	115	63.5	42	108	< 0.001

*Mann–Whitney U-test.

**Figure 3:**
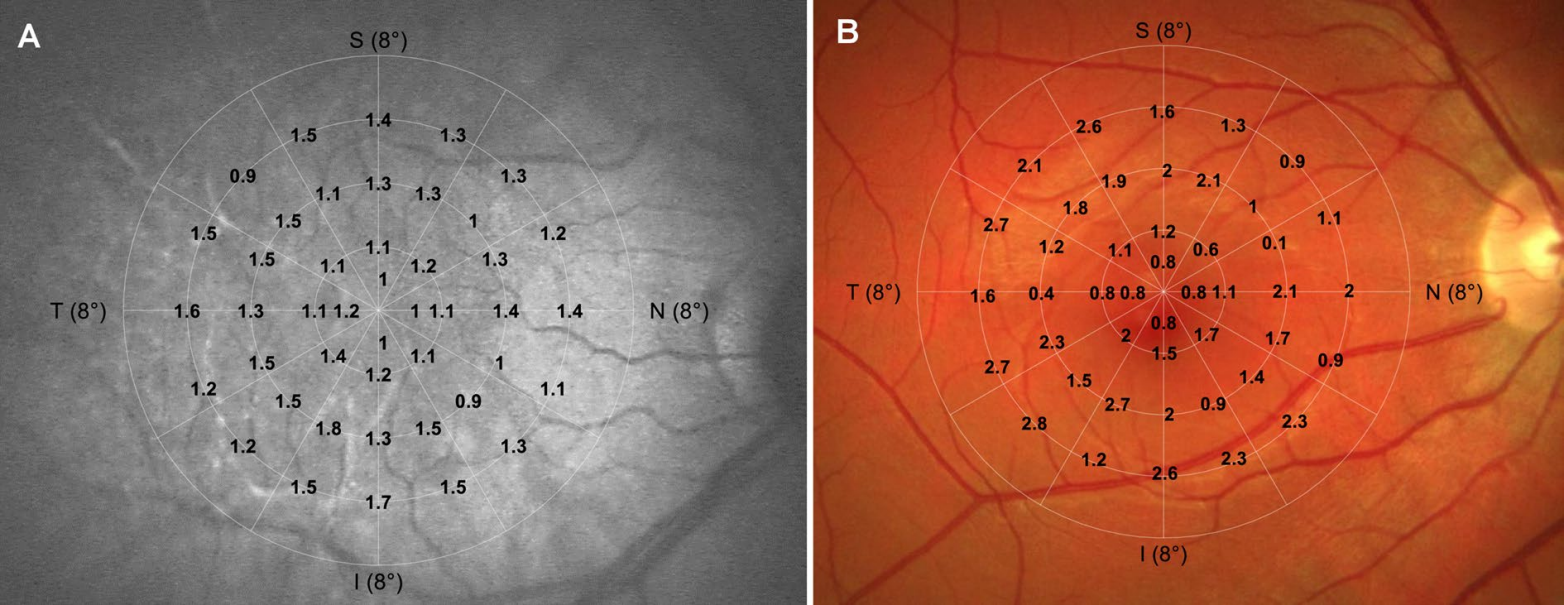
The absolute difference between IR- (**A**) and visible-light (**B**) sensitivity measured at each retinal location. The test grid was projected on an exemplary fundus image obtained with an SLO (**A**) and a standard photographic (**B**) technique.

**Figure 4:**
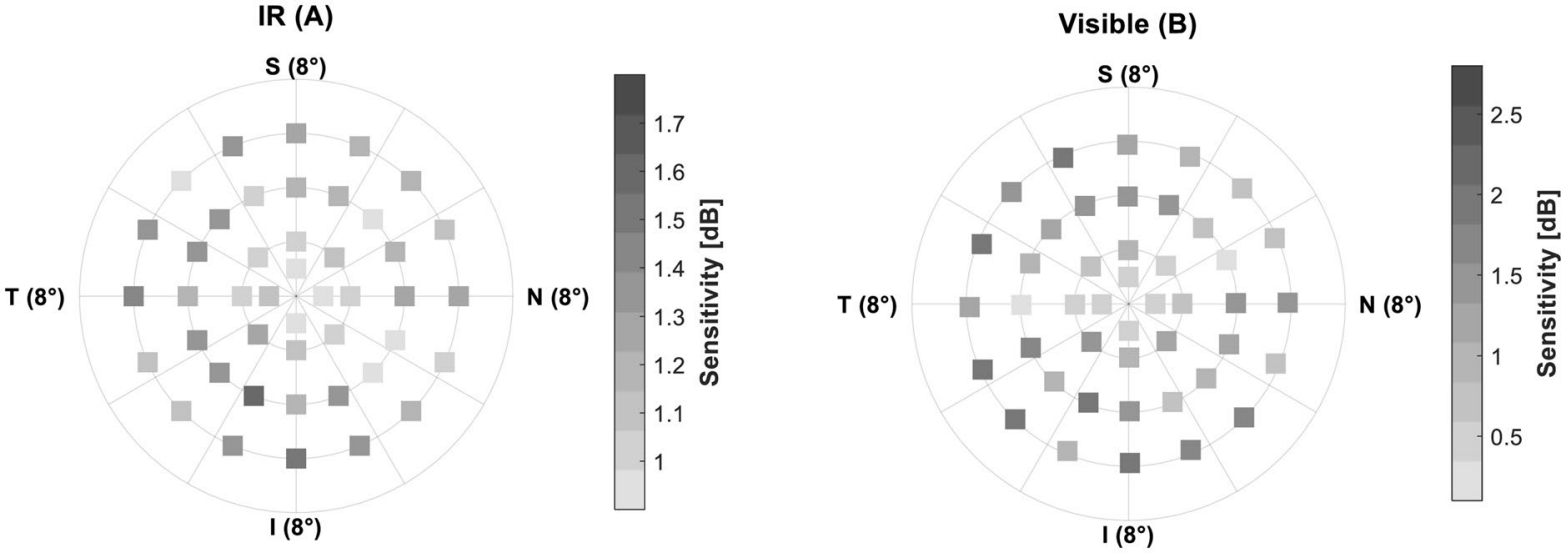
Grayscale-coded comparison of the absolute difference between IR (**A**) and visible (**B**) sensitivity. The color-coding was set according to the minimum and maximum values with a gradual change of gray shades by half of the standard deviation.

Both the visible- and IR-light sensitivity were decreased in the glaucoma patients compared to the controls; however, only in the latter test, the difference was statistically significant. Although the median sensitivity to visible light was 17.7 (11.6 to 20.0) dB in the healthy eyes and 17.0 (6.9 to 20.0) dB in the ones with glaucoma, the *p*-value was 0.07. The IR-sensitivity comparison yielded the median sensitivity of 10.9 (8.2 to 13.0) dB and 9.8 (6.5 to 13.1) dB in the healthy and glaucoma population, respectively (*P* = 0.003). Figure 3 shows the absolute difference of IR- (A) and visible-light (B) sensitivity at each tested locus with a grayscale-coded visualization presented in Fig. 4. In both tests, the difference minimally increases from the center to the periphery of the test grid. Figures S1 and S2 (Supplementary Information) contain the original sensitivity values for each condition and group.

**Figure 5:**
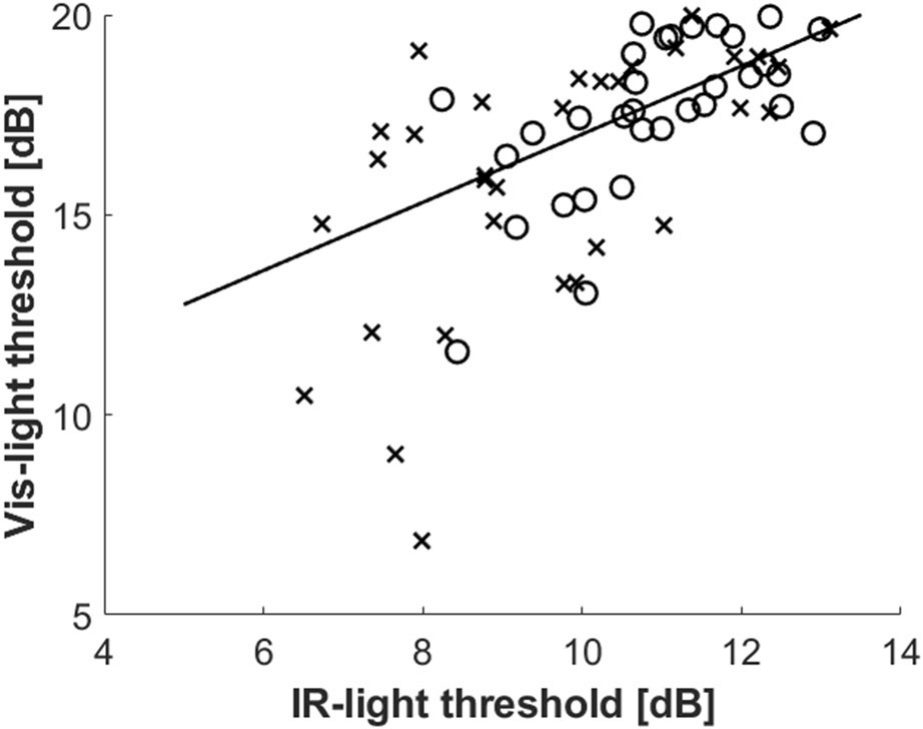
The comparison between the average visible- and IR-light sensitivity assessed in control (circles) and glaucoma (crosses) populations. The solid line refers to the quantile regression of the data.

Figure 5 demonstrates the correlation between sensitivity measured in visible and IR light. We found a high and significant correlation between the two methods (ρ = 0.66, *P* < 0.001). However, the agreement appears to be worse in patients with substantially decreased sensitivity.

**Figure 6:**
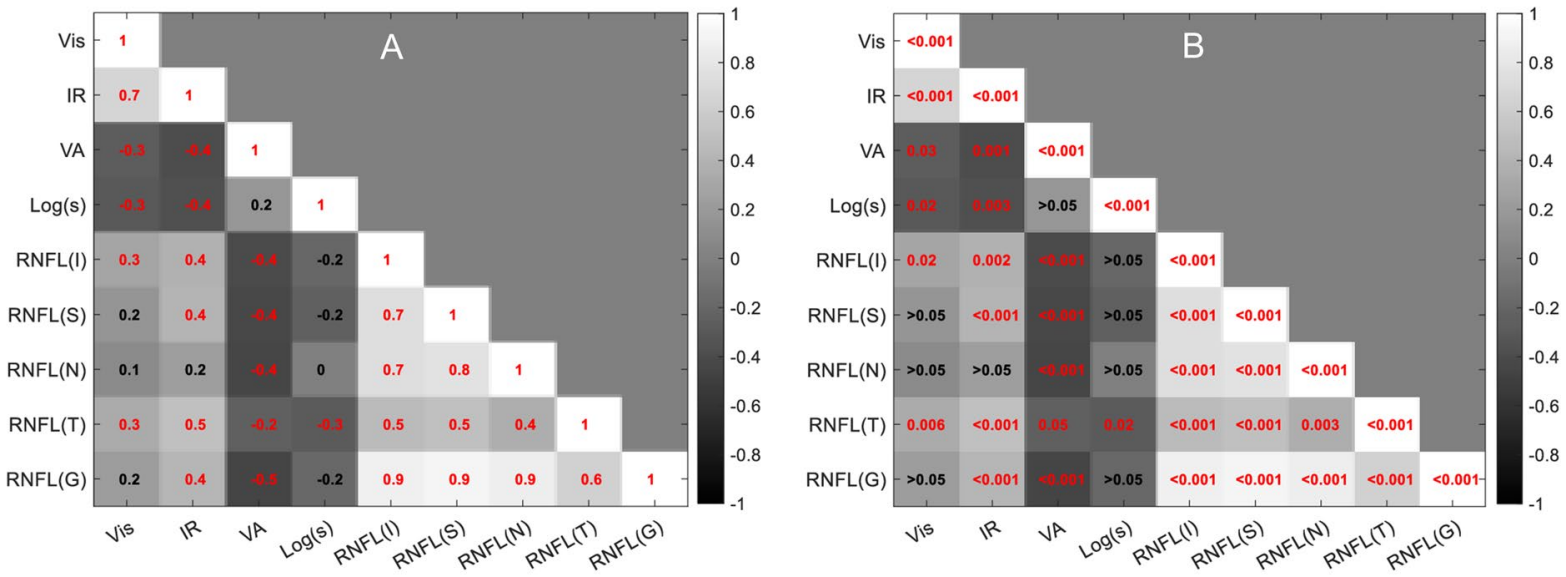
The correlation matrix for the study parameters and the corresponding Spearman's rank correlation coefficient (ρ) (**A**) and *p*-values (**B**) with statistically significant correlations marked in red. The color scale bar indicates the ρ level. Vis = visible-light sensitivity, IR = IR-light sensitivity; log(s) = logarithm of the straylight parameter; RNFL = retinal nerve fiber layer, I = inferior; S = superior; N = nasal; T = temporal; G = global.

The correspondence between the objective and functional parameters was summarized in a matrix and presented in Fig. 6. The visible and IR sensitivity demonstrated a significant correlation with VA and log(s). IR-light sensitivity was also significantly correlated with all RNFL metrics but one, as compared to only one detected with the visible-spectrum device. There was no correlation between logs(s) and VA, but the RNFL parameters' correlation coefficient was the highest, ranging from 0.33 to 0.92. VA (logMAR) demonstrated a significant correlation with all the RNFL parameters with ρ values from − 0.2 to − 0.5.

Figure S3 (Supplementary Information) presents the distribution of the studied parameters and their comparison between the two groups.

## Discussion

We demonstrated that glaucomatous eyes have significantly decreased sensitivity to IR light compared to eyes in an age-matched control group. The eye's IR perception could act as a new functional parameter in detecting neuropathy more effectively in glaucoma diagnosis than using (first-generation) visible-light microperimetry. Although both approaches showed a good agreement, the two-photon device demonstrated a higher correlation with the glaucoma patients' changed morphology.

The application of visible-light microperimetry to detect glaucomatous retinal sensitivity was studied by Oztürk et al. in patients with open-angle glaucoma and their results were compared with healthy controls^5^. They measured visibility thresholds using the MP-1 microperimeter and found the mean (± standard deviation) value of their glaucoma population was 16.23 ± 2.28 dB, while it was 17.79 ± 1.09 dB for the controls. Those are close to the values that we found: our glaucoma patients' average was 16.0 ± 3.3 dB, and the control was 17.6 ± 2.0 dB. Please note that we report the median (range) in describing our results because our data’s lack of normal distribution. Despite the difference between the study and control means, our comparison was not statistically significant, and that is in disagreement with the conclusions of Oztürk's group^5^. Klamann et al. also could not find visible-light sensitivity to differ between their glaucoma and healthy population^9^. The lack of significant difference in our study might be due to the higher spread of the values and our use of a non-parametric test. Still, the absolute values were comparable, indicating reliable reproducibility of the visible-range microperimetry data in glaucoma and normal populations. However, the dynamic range of the MP-1 might have been a limiting factor in this comparison with the two-photon device. Future research could examine whether a new generation of microperimeter (for example, the MP-3 model) with an extended dynamic range could prove more sensitive in discriminating between glaucomatous and normal subjects.

Oztürk and colleagues reported a significant correlation between standard automated perimetry (SAP) and microperimetry with the Pearson correlation coefficient of 0.46^5^. In a subsequent study, Rao et al. compared retinal sensitivity in glaucoma and healthy eyes measured also using SAP and microperimetry, and they found over time a significant sensitivity loss during the progressive course of glaucoma^10^. They noted the relationship between the visibility threshold and the thinning of the inner plexiform layer of macular ganglion cells was independent of the type of perimetric method^10^. Lima et al. took a similar approach and found a strong correlation of the visibility thresholds in outcomes obtained with the SAP and SLO-based devices, with the coefficient of determination (R^2^) ranging from 0.68 to 0.85 in four different quadrants^7^. Lima and colleagues concluded that microperimetry might better detect morphological changes at the retina, which was confirmed by assessing macular thickness in OCT imaging^7^. We found a comparable correlation between IR- and visible-light microperimetry, indicating that the former approach may be considered an alternative for central visual field screening when assessing glaucomatous patients. One that is free of a ceiling-effect observed by Steinberg et al. with visible-light microperimetry^25^. The distinction should be made, however, that the two devices operate at different light-intensity levels as the maximum stimulus intensity was 127 $\frac{cd}{{m}^{2}}$ (400 abs), but for the two-photon device, it was 400 µW. The conversion from radiometric to photometric units for an IR-light source is impossible given the latter’s reliance on the human-eye sensitivity data, which does not cover the IR range. Furthermore, retinal sensitivity to two-photon 1045-nm stimulus is 86 dB lower than if a 522.5-nm (green) beam in a standard (single-photon) perception is used^16^, which needs to be taken into account while interpreting the current results.

Our study indicates that IR-light sensitivity could be a better predictor of glaucomatous neuropathy than visible-light (first-generation) microperimetry. Klamann et al. reported an evident correlation between standard-microperimetry results and the RNFL thinning only in the inferior-temporal quadrant^9^. We confirmed this observation; we found the correlation between visible-light sensitivity and the RNFL thickness was significant only in the temporal quadrant. By contrast, the two-photon device's correlation with OCT morphological parameterswas significant for all but one (nasal). Takagishi et al. demonstrated that the RNFL is a precise measure comparable to SAP in detecting glaucomatous neuropathy^8^. Monsalve et al. confirmed those results with two standard-perimetry systems^11^. How two-photon sensitivity compares with SAP in the discrimination between glaucomatous and healthy eyes, particularly in the early detection of glaucomatous neuropathy, ought to be addressed in a clinical study. In such a study, however, a point-wise analysis rather than an average value should be used to allow for a more localized examination.

Since Palczewska et al.^15^ demonstrated the principle of two-photon absorption by photoreceptors; and provided evidence for color perception through IR-light stimulation, our knowledge of two-photon vision has improved through several studies. Rumiński et al., in an extensive laboratory examination of human subjects, compared 1045- and 522.5-nm sensitivity measured by one device. A non-linear crystal in the process of second-harmonic generation produced the green stimulus^16^. Their comparison confirmed an excellent test–retest outcome of the laser-driven microperimetry, but the IR-light approach demonstrated higher repeatability than the one with green light^16^. IR-sensitivity reported by Rumiński et al. (i.e.,12.4 µW for rod plateau) corresponds to 15.1 dB of the current study, and is much higher than we found in our control group. We can account for this in the differences in the testing conditions, as we made measurements with dim white-light background illumination and after 5-minunte dark adaptation, which permits lower retinal sensitivity. In our recent study on IR sensitivity in diabetic retinopathy eyes, which we conducted to test the feasibility of IR-light testing in a clinical setting^17^, we obtained 15.5 ± 1.3 dB in dark-adapted healthy eyes. This result is close to the level reported by Rumiński et al. We confirmed that the two-photon device could be applied successfully to detect and monitor diabetic retinopathy. We found a significant decrease in IR-light sensitivity in the diabetic populations^17^. Our current investigation is the first to assess IR vision in glaucoma patients, also demonstrating compromised retinal sensitivity. However, we recruited only patients with advanced glaucoma and this may explain a high correlation between the RNFL loss and VA and a significant VA difference between the two groups, given that VA is only affected at the later stage of the disease progression^26^. Besides, this may also explain the found correlation of VA with (average) IR- and visible-light sensitivity. Further research is needed to determine how the IR-light perception changes with glaucoma severity and type. Also, implementing a point-wise comparison might be advantageous once we have established the normative data for older age groups.

IR sensitivity is a new functional parameter, and it is only recently that its distribution was determined in a younger population (≤ 60 years of age)^18^. In previous research to assess the age-dependencyof IR vision, we studied subjects ranging from 21 to 70 years of age^18^. The group was assessed after a 30-min dark adaptation and with no background illumination, which contrasts with our glaucoma study protocol. In the first study, we found an IR-sensitivity decline of 0.18 dB per decade in the healthy cohort. We concluded that lens turbidity (straylight) had minimal effect on IR-light sensitivity as we did not find a significant correlation between these parameters. In the current glaucoma study, we observed a decline of IR-light sensitivity with increasing straylight. One explanation for this may be that the median age of our glaucoma and control groups was 65.4 years compared to 44.1 years when we studied age dependency^18^, which in turn may explain a higher straylight level (1.10 log[s]) found in the glaucoma study compared to 0.92 log[s] in the earlier study. However, one may also point to other confounding factors, the inclusion of the dim background or to an age-related increase of higher-order aberrations^27^. Nevertheless, these results indicate that increased straylight may affect IR-light sensitivity measurements. Still, the impact of lens opacification is 2.7 times lower on IR than on visible light sensitivity^16^. More research is needed to quantify straylight effects on IR-light sensitivity of cataract patients before and after intraocular lens implantation.

Two-photon technology is not yet widely accessible. We used a research tool equipped with measurements strategies that are not yet well developed. Although this device uses a standard staircase method, one cannot change the dynamic of approaching the threshold. Thus, the examination time is prolonged, as one makes an effort to maintain high-precision measurements despite the equipment’s limitations. The test procedure is of relatively long duration (spanning from 10 to 15 min) compared to a session using one of the commercially available microperimeters, utilizing modern testing algorithms. The longer the procedure, the more likely it is to fatigue patients, which can impact the results. Another aspect, as Montolio et al. have shown, is the subjective character of (micro)perimetry, which many factors can influence^28^. Nevertheless, standard automated perimetry will remain the preferred approach in glaucoma management given its high reliability in most glaucoma patients, who become accustomed to performing sensitivity testing^29^, and for providing the link between the morphological and functional changes^6^. We recognize the research equipment we used needs to undergo further development before being considered ready for routine clinical implementation.

An additional restriction to the transfer from the laboratory to the examination room of this two-photon technology is the cost of the femtosecond light source, a solid-state laser. At present, it is close to the price of the latest generation microperimeters. A recent finding where the device's laser could be replaced with a fiber IR laser may prove more cost-effective, which could accelerate the commercialization of this technology^19^.

In conclusion, IR-light sensitivity may provide in the future a new tool for detecting glaucoma. The two-photon setup is at the development stage, but we demonstrated that it outperformed standard (first-generation) microperimetry showing a better correlation between retinal sensitivity (functional effect) and thinning of the RNFL (morphological changes). Further development of this research device is needed to overcome the current limitations and increase its applicability in clinical practice.

## Supplementary Information

Supplementary Information 1.

## Data Availability

The datasets generated during and/or analyzed during the current study are available from the corresponding author on request.
