# Supplementary material for: Infrared- and white-light retinal sensitivity in glaucomatous neuropathy
Source: Sci Rep. 2022 Feb 4;12:1961. doi: 10.1038/s41598-022-05718-6 (PMC8816930; doi:10.1038/s41598-022-05718-6)
Supplement: Supplementary file 1 — Supplementary Information 1. [file 41598_2022_5718_MOESM1_ESM.pdf]

Supplementary Information

Glaucoma (A)

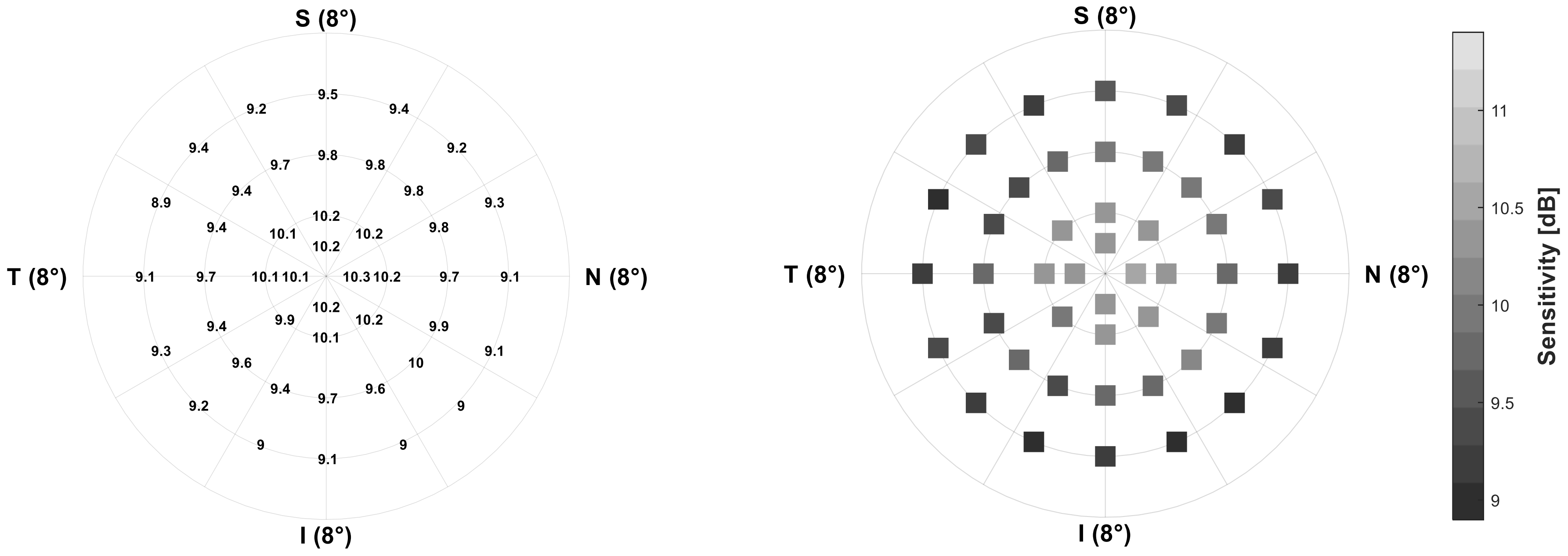

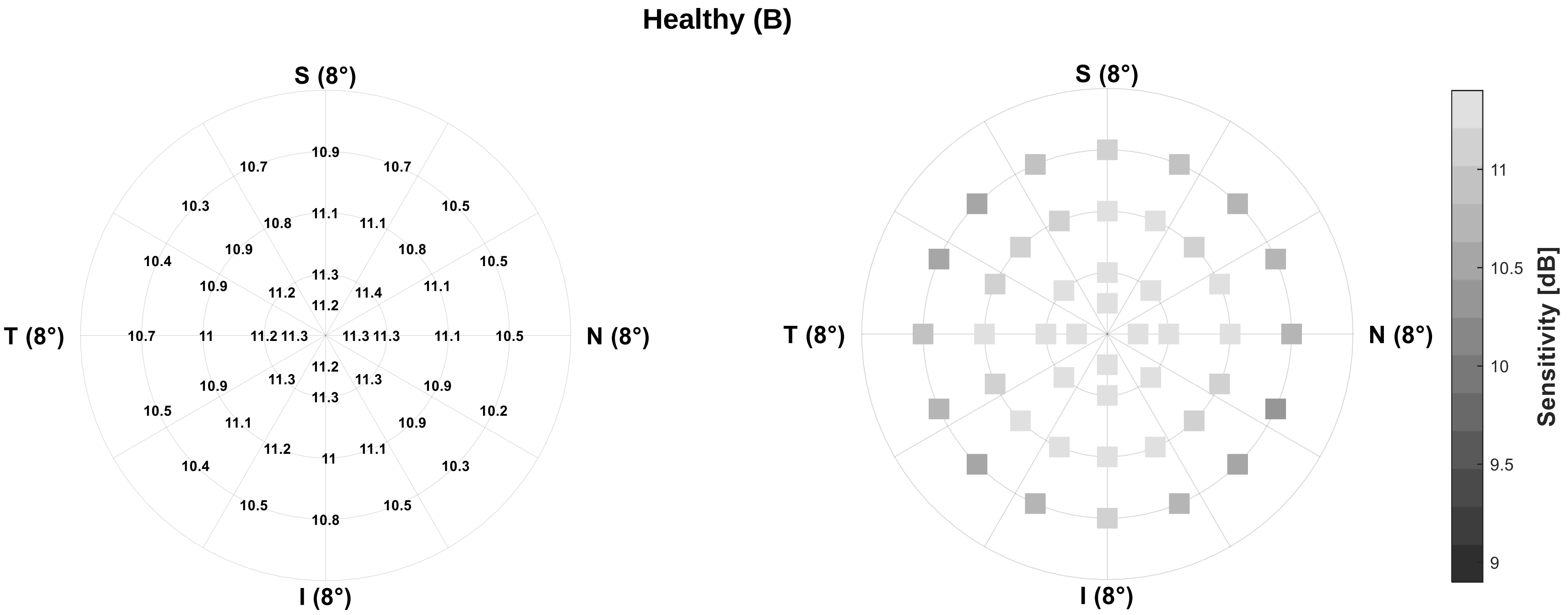

Figure S1. The average retinal-sensitivity values in decibels (left panels) and color-coded sensitivity levels (right panels) of patients with glaucoma (A) and healthy controls (B). Measurements were performed in infrared (1045 nm) light. The color-coding was set according to the minimum and maximum values of the entire population with a gradual change of gray shades by half of the standard deviation.

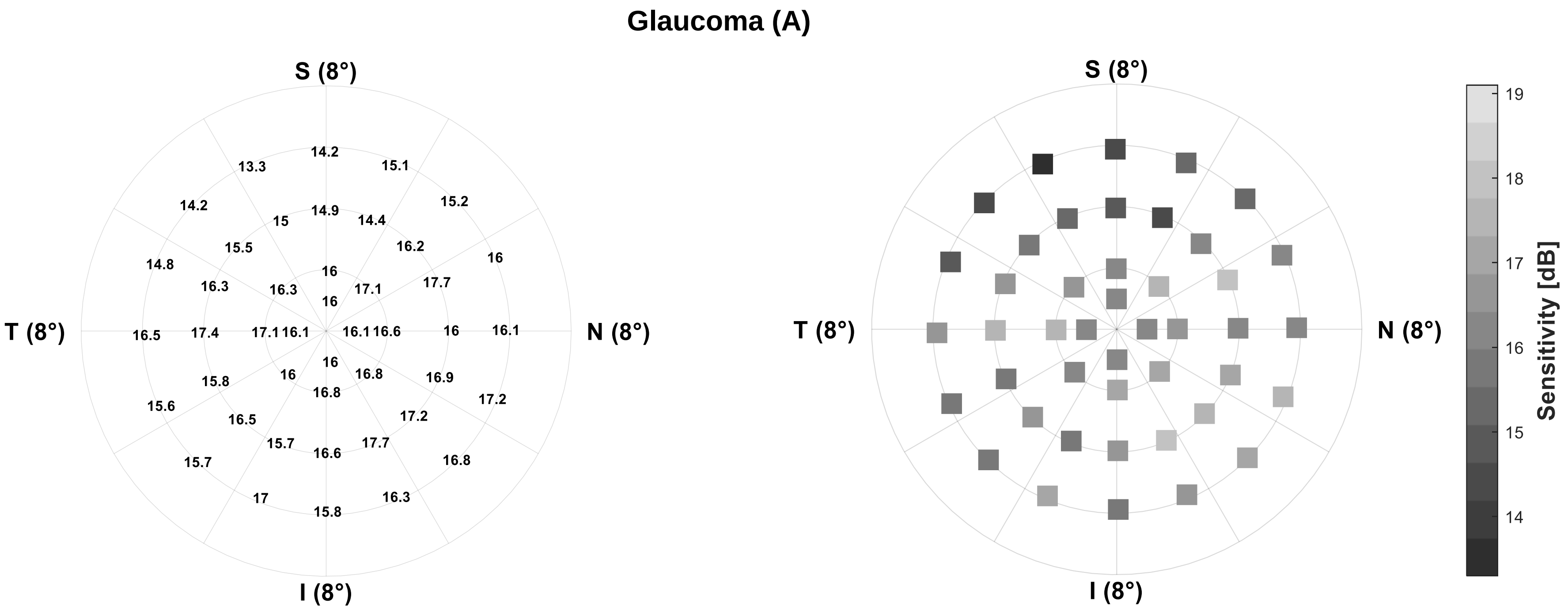
Glaucoma (A)
S (8°)
T (8°)
N (8°)
I (8°)
Sensitivity [dB]
14
15
16
17
18
19

Healthy (B)

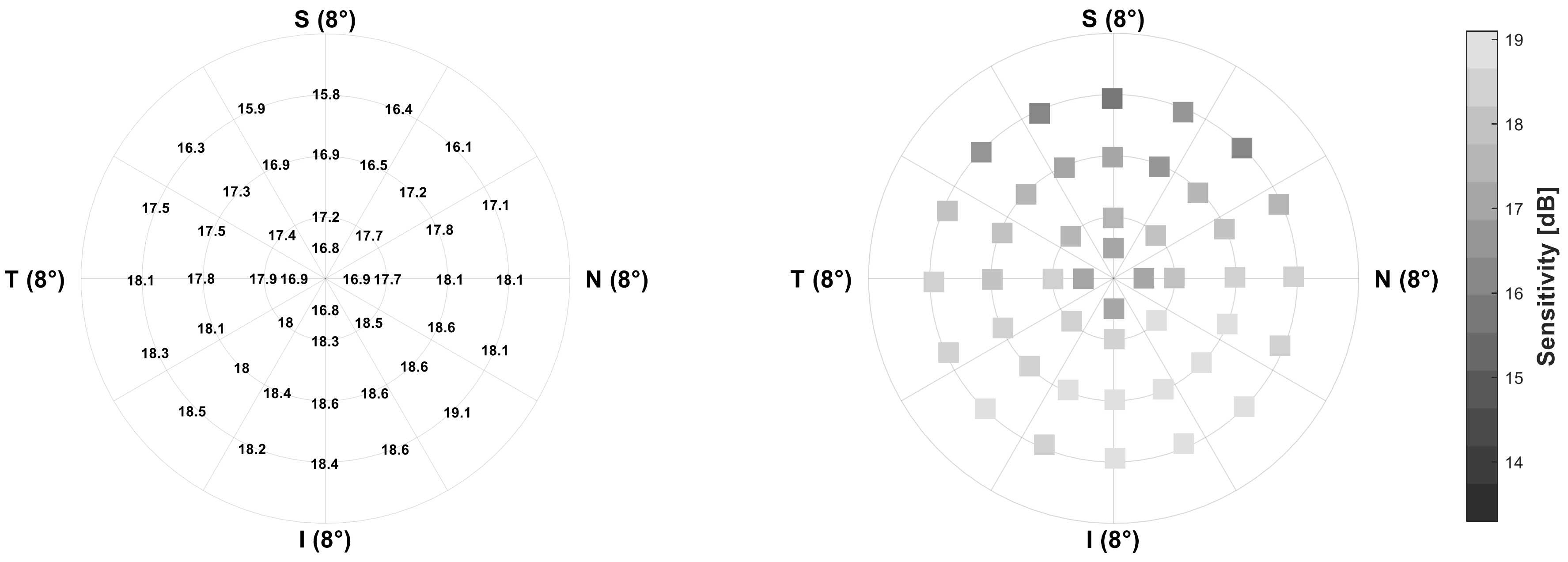

Figure S2. The average retinal-sensitivity values in decibels (left panels) and color-coded sensitivity levels (right panels) of patients with glaucoma (A) and healthy controls (B). Measurements were performed in visible (white) light. The color-coding was set according to the minimum and maximum values of the entire population with a gradual change of gray shades by half of the standard deviation.

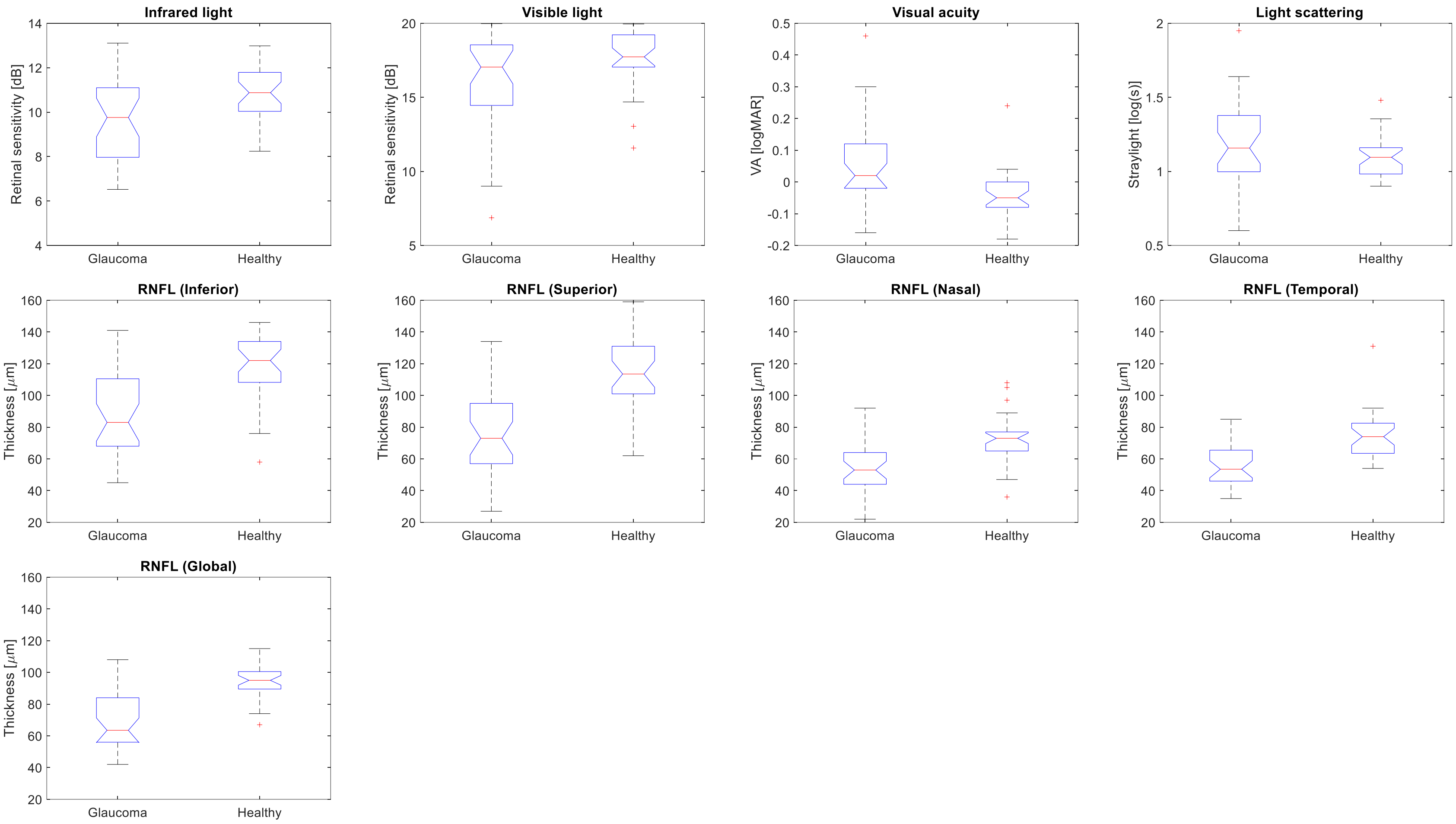

Figure S3. The box plots presenting the distribution of the studied visual parameters in the glaucoma and control groups. RNFL = retinal nerve fiber layer. Middle lines = median; box edges = the 25th (bottom) and 75th (top) percentiles; whiskers = adjacent values; crosses = outliers.
